# Molecular identification of endophytic fungi in lawn grass (*Axonopus compressus*) and their pathogenic ability

**DOI:** 10.1038/s41598-023-31291-7

**Published:** 2023-03-14

**Authors:** Nurul Farizah Azuddin, Mohamad Syahril Mohamad Noor Azmy, Latiffah Zakaria

**Affiliations:** School of Biological Sciences, Universiti Sains Malaysia, 11800 USM Penang, Malaysia

**Keywords:** Fungal pathogenesis, Parasitism

## Abstract

Lawn grass (*Axonopus compressus*) is a widely distributed grass species from the family Poaceae that is ubiquitous in Malaysia. We isolated endophytic fungi from the leaves of *A. compressus* and molecularly identified them as *Fusarium parceramosum*, *Colletotrichum siamense*, *C. gigasporum*, *C. endophyticum*, *Curvularia lunata*, *Stagonospora bicolor*, *Calonectria gracilis*, and *Albifimbria verrucari*. These fungal endophytes are considered host generalists, as they have been isolated from other plants and have also been reported to be latent plant pathogens. We tested the pathogenicity of selected endophytic fungal isolates on *A. compressus* leaves, chili (*Capsicum annum*), and tomato (*Solanum lycopersicum*), and found that they were pathogenic to wounded *A. compressus* leaves with low to moderate virulence, and several were pathogenic to wounded and unwounded chili and tomato fruits. This indicated that the endophytes could infect both vegetable fruits with low to very high virulence. Pathogenicity tests demonstrated that endophytic fungi from the leaves of *A. compressus* can become pathogenic and infect the host and other plant species. The findings also indicated that leaves of *A. compressus* may harbor pathogens with latent ability that can become active due to changes in environmental conditions, thereby disrupting the balance between host-endophyte antagonism.

## Introduction

*Axonopus compressus* (Sw.) P. Beauv. is a perennial, short-spreading grass that forms creeping stems with long stolon spread by aboveground runners and roots at the nodes^1^. It is also called lawn grass, tropical carpet grass, blanket grass, broadleaf carpet grass, and savannah grass. Although *A. compressus* originated in the Americas in the region from the southern USA to Argentina, the grass is distributed in many tropical and subtropical countries, including Malaysia^2^.

In Malaysia, *A. compressus* is used as lawn grass, in turf gardens for landscaping, and on sports fields^3^. Other uses include pastures for animal grazing, ground cover in oil palm and rubber plantations, and controlling soil erosion^1,4^. It is considered a weed when dense growth of the grass surrounds and covers young crops. Additionally, *A. compressus* has medicinal values and is used in antimalarial, antidiabetic, and hemorrhoid treatment^5^.

Endophytic fungi residing in grasses were first studied when Guerin^6^ and Freeman^7^ detected mycelia within healthy seeds of the ryegrass *Lolium temulentum*. Decades later, studies on endophytic fungi in grasses focused on pasture grasses used as animal feed, especially after endophytic *Neotyphodium* and the teleomorph *Epichloe* were found to produce alkaloids that were toxic to animals^8,9^. Endophytes have been ubiquitously detected in plants other than grasses^10,11^.

Endophytic fungi can penetrate and reside internally in plant tissues without causing any damage to the host for at least a part of their life cycle^12^. Plants and fungi interact in various manners ranging from mutualism to antagonism. Endophytes can be mutualists, latent saprophytes, or latent pathogens to plants^11^. During the latent phase, endophytic fungi can become pathogenic, altering host physiology and rendering it prone to infection. This transition may be induced by biotic (host plant resistance level) and abiotic factors (pH, temperature, or humidity)^13^. The host plant can also act as an alternative host for plant pathogens. Moreover, many endophytic fungi have a wide range of hosts and can infect various plant species^14,15^.

There are many reports of endophytic fungi residing in grass species, particularly in temperate countries. Various species of endophytic fungi have been reported in Timothy grass (*Phleum pratense*) and ryegrass (*Lolium perenne*)^16^, pasture grass (*Brachiaria* sp.)^17^, Italian ryegrass (*Lolium multiflorum*)^18^, Asian crabgrass (*Digitaria bicornis*), and yellow watercrown grass (*Paspalidium flavidum*)^19^. In Malaysia, endophytic *Fusarium* spp. have been recovered from several grass species^20,21^.

Despite many reports of endophytic fungi, their occurrence in grasses in Malaysia has received little attention. There is inadequate data and information regarding endophytic fungi in grasses and their pathogenicity. Therefore, we aimed to isolate and identify endophytic fungi from lawn grass (*Axonopus compressus*) and determine their pathogenic ability towards its host, as well as two important vegetable fruits, chili (*Capsicum annum*) and tomato (*Solanum lycopersicum*).

## Results

### Morphological and molecular identification

A total of 42 isolates of endophytic fungi, consisting of six genera and eight species, were recovered from 20 leaf tissues of *A. compressus*. Based on molecular identification and phylogenetic analysis using several markers, the endophytic fungi recovered from the leaves of *A. compressus* were identified as the *Fusarium solani* species complex (n = 14), *Colletotrichum siamense* (n = 8), *C. gigasporum* (n = 2), *C. endophyticum* (n = 2), *Curvularia lunata* (n = 7), *Stagonospora bicolor* (n = 5), *Calonectria gracilis* (n = 3), and *Albifimbria verrucaria* (n = 1). A BLAST search of the isolates showed 98–100% similarity with the sequences in GenBank (Supplementary Table 1 online). For the *Fusarium solani* species complex, the BLAST search against Fusarium-ID showed similarity with several species within the species complex, of which the isolates had 97–99% similarity with *Fusarium liriodendri, Fusarium parceramosum,* and* Fusarium perseae* (Supplementary Table 2 online).

**Figure 1:**
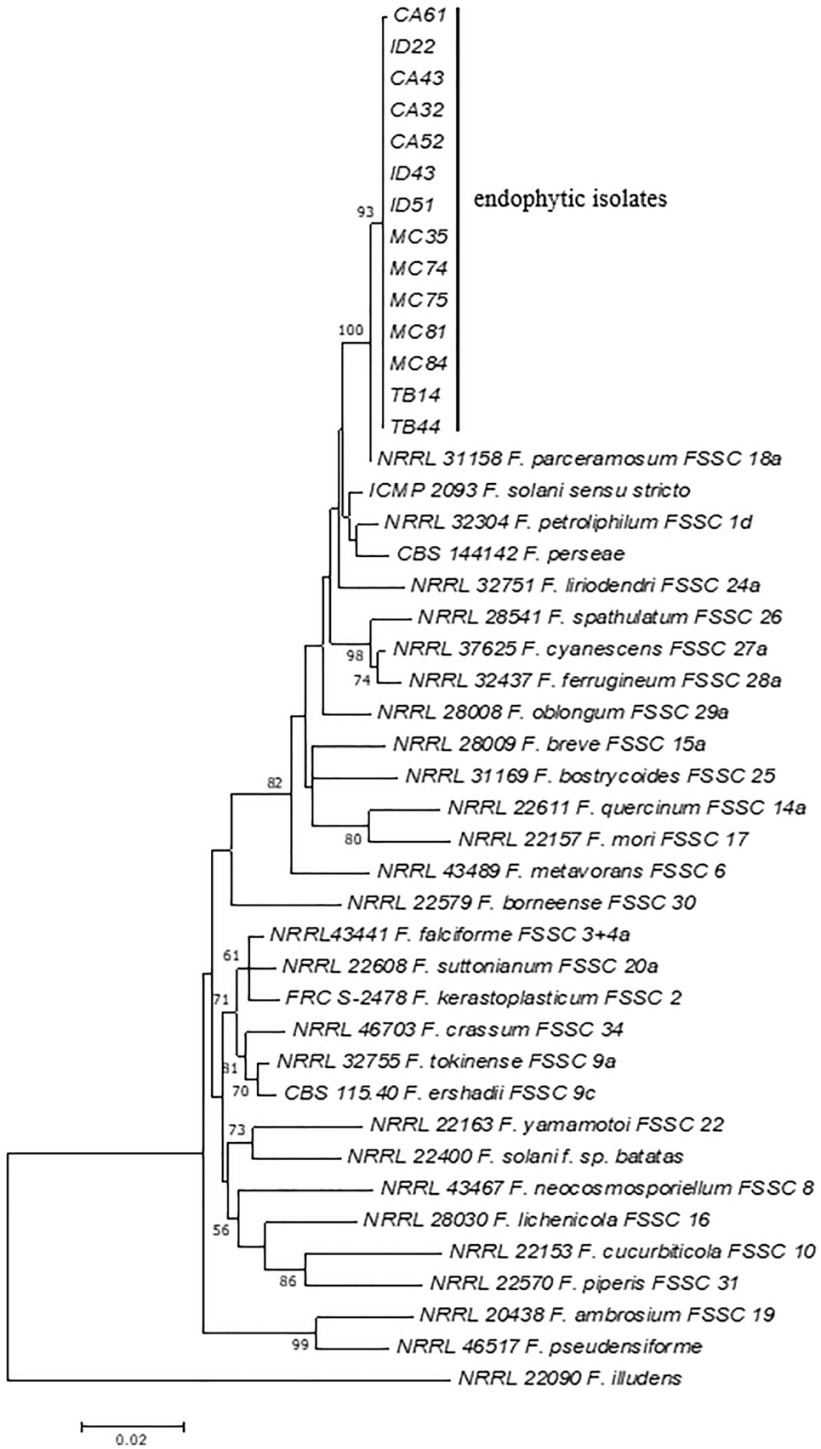
Maximum likelihood tree inferred from combined sequences of TEF-1α, and RPB2 of endophytic *F. solani* species complex isolated from *A. compressus* leaves.

Based on the phylogenetic analysis of individual TEF-1α sequences (Supplementary Fig. 1 online) and combined TEF-1α and RPB2 sequences (Fig. 1), endophytic isolates of the *F. solani* complex from *A. compressus* were clustered together in the main clade with *F. parceramosum,* indicating that the isolates have high sequence similarity with *F. parceramosum.* Thus, the isolates were phylogenetically identified as *F. parceramosum.*

**Figure 2:**
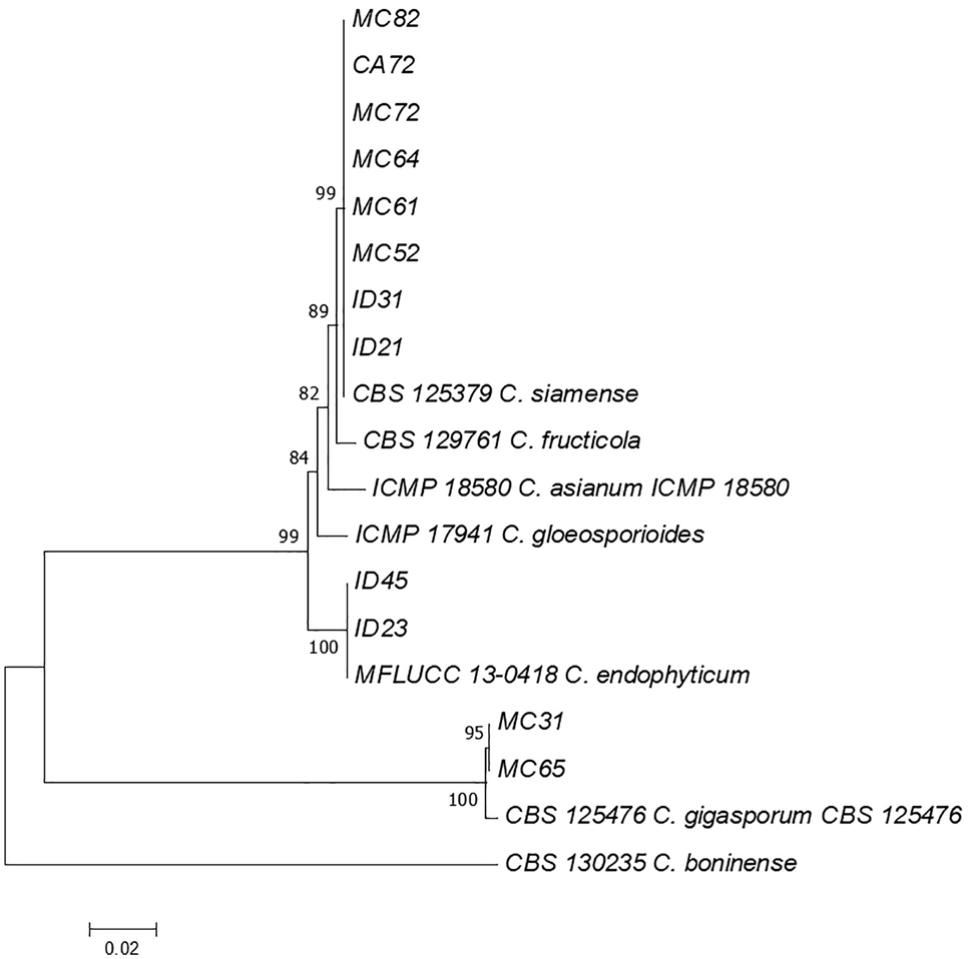
Maximum likelihood tree inferred from combined sequences of ITS, GAPDH, β-tubulin, and ACT of endophytic *Colletotrichum* spp. isolated from *A. compressus* leaves.

Three *Colletotrichum* species*, C. siamense* (n = 8), *C. gigasporum* (n = 2), and *C. endophyticum* (n = 2), were identified based on a combination of ITS, GAPDH, β-tubulin, and ACT sequences. These three *Colletotrichum* species are members of the *C. gloeosporioides* species complex. Phylogenetic analysis showed that the three *Colletotrichum* species clustered with their epitype strains (Fig. 2).

**Figure 3:**
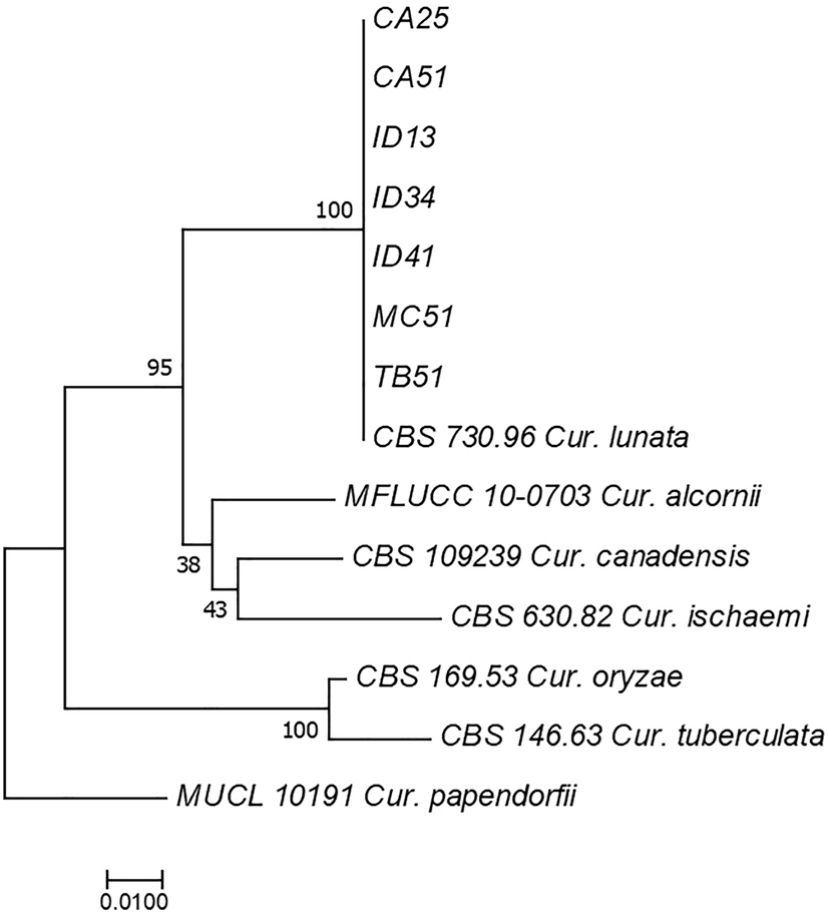
Maximum likelihood tree inferred from combined sequences of ITS and GAPDH of endophytic *C. lunata* from *A. compressus* leaves.

**Figure 4:**
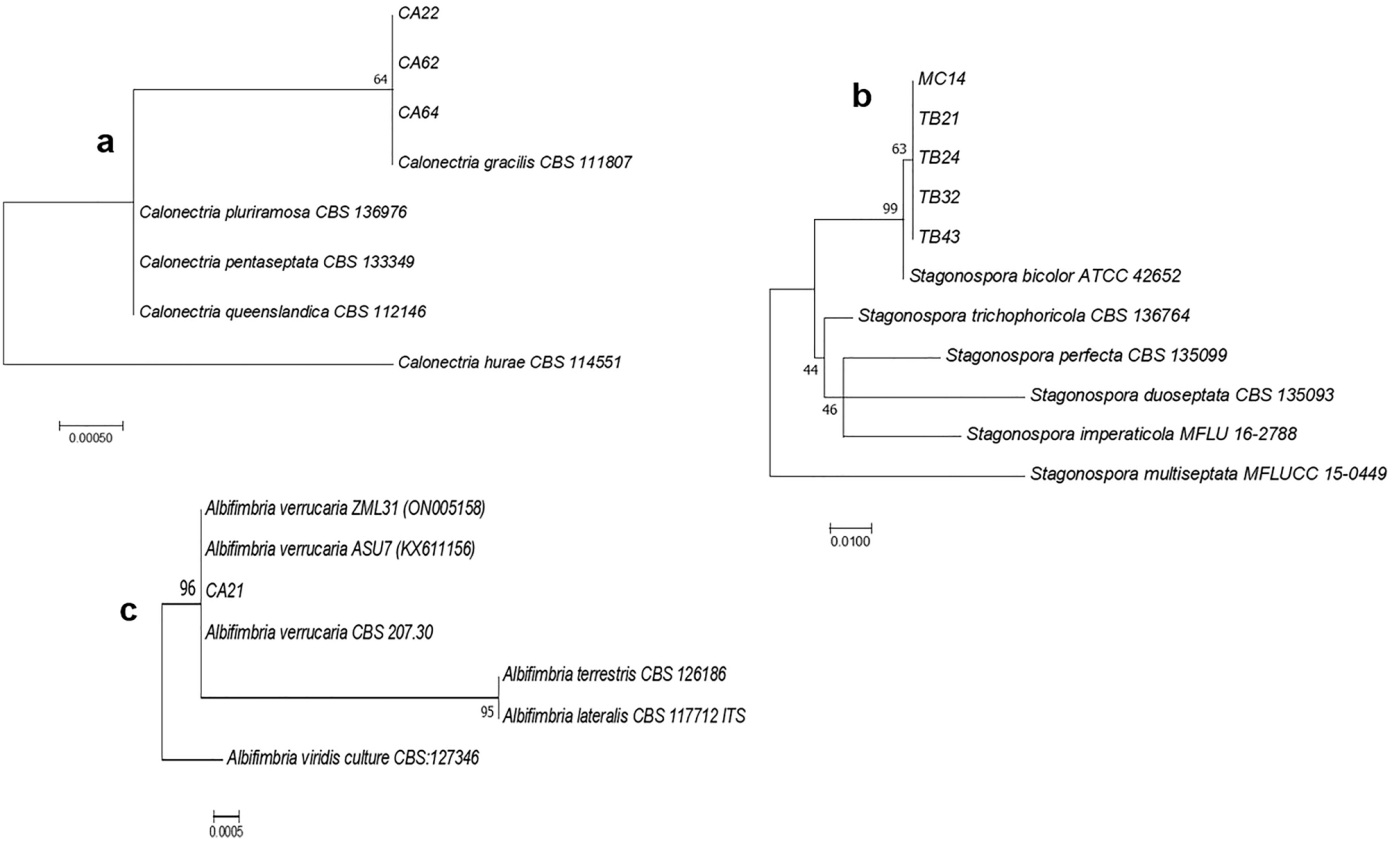
Maximum likelihood trees inferred from ITS sequences of endophytic (**a**) *C. gracilis* (**b**) *S. bicolor* and (**c**) *A. verrucaria* isolated from *A. compressus* leaves.

Seven isolates of *C. lunata* were identified using the ITS and GAPDH sequences (Supplementary Table 1, Fig. 3), whereas three isolates of *C. gracilis* (Supplementary Table 1, Fig. 4a), five isolates of *S. bicolor* (Supplementary Table 1, Fig. 4b), and one isolate of *A. verrucaria* (Supplementary Table 1, Fig. 4c) were identified based on the ITS sequences. Phylogenetic analysis demonstrated that the same species could be grouped according to their epitype or reference strains.

### Pathogenicity test

A pathogenicity test of 26 selected isolates of endophytic fungi from *A. compressus* showed that they were able to cause infection on wounded leaves with a low to moderate degree of virulence (Table 1). Ten endophytic fungal isolates were non-pathogenic on wounded chili fruits: *C. lunata* (ID34, MC51, CA25, and TB51), *C. gracilis* (CA22 and CA64), *S. bicolor* (MC14, TB21, and TB43), and *A. verrucaria* (CA21) (Table 1). Isolates of *C. gracilis* (CA22 and CA64) and *S. bicolor* (MC14, TB21, and TB43) were also non-pathogenic to tomato fruits (Table 1).

**Table 1:** Pathogenicity of endophytic fungi on wounded *A. compressus* leaves, chili, and tomato on 7 day after inoculation.

Isolate	Grass leaves			Chilli			Tomato		
	Lesion length (cm)	Disease severity (%)	Degree of virulence	Lesion length (cm)	Disease severity (%)	Degree of virulence	Lesion length (cm)	Disease severity (%)	Degree of virulence
*C. endophyticum* (ID23)	0.1–0.1	33.33^c^	Low	0.5–1.0	26.67^c^	Low	2.0–2.5	60.00^g^	Moderate
*C. siamense* (ID31)	0.1–0.2	33.33^c^	Low	0.3–0.5	20.00^b^	Low	– 2.1	53.33^f^	Moderate
*C. siamense* (MC52)	0.1–0.1	33.33^c^	Low	0.2–0.5	20.00^b^	Low	0.9–1.1	33.33^d^	Low
*C. siamense* (MC64)	0.1–0.1	33.33^c^	Low	0.5–1.0	20.00^b^	Low	0.2–0.8	20.00^b^	Low
*C. siamense* (CA72)	0.1–0.2	33.33^c^	Low	1.0–1.3	40.00^d^	Moderate	0.2–0.5	20.00^b^	Low
*C. gigasporum* (MC31)	0.1–0.1	33.33^c^	Low	1.5–3.0	60.00^g^	Moderate	0.1–0.1	20.00^b^	Low
*C. gigasporum* (MC65)	0.1–0.1	33.33^c^	Low	0.2–0.3	20.00^b^	Low	0.1–0.1	20.00^b^	Low
*C. endophyticum* (ID45)	0.1–0.1	33.33^c^	Low	0.5–3.0	46.67^e^	Moderate	1.0–2.0	53.33^f^	Moderate
*F. parceramosum* (ID22)	0.1–0.1	33.33^c^	Low	3.7–4.0	93.33^j^	Very high	0.7–1.4	33.33^d^	Low
*F. parceramosum* (ID51)	0.1–0.2	33.33^c^	Low	0.5–3.0	46.67^e^	Moderate	0.2–0.8	20.00^b^	Low
*F. parceramosum* (MC35)	0.1–0.2	33.33^c^	Low	3.7–4.5	93.33^j^	Very high	0.4–1.4	26.67^c^	Low
*F. parceramosum* (MC81)	0.0–0.2	22.22^b^	Low	2.7–3.0	66.67^h^	High	0.8–1.1	40.00^e^	Moderate
*F. parceramosum* (CA52)	0.0–0.0	22.22^b^	Low	4.0–5.0	100^ k^	Very high	1.2–1.8	40.00^e^	Moderate
*F. parceramosum* (CA61)	0.0–0.3	33.33^c^	Low	4.0–4.5	100^ k^	Very high	0.4–0.5	20.00^b^	Low
*F. parceramosum* (TB14)	0.1–0.1	33.33^c^	Low	2.0–3.5	73.33^i^	Very high	0.2–1.1	26.67^c^	Low
*F. parceramosum* (TB44)	0.1–0.2	33.33^c^	Low	1.5–2.3	53.33^f^	Moderate	1.4–1.9	40.00^e^	Moderate
*C. lunata* (ID34)	0.2–0.3	33.33^c^	Low	0	0^a^	Avirulence	0.1–0.1	20.00^b^	Low
*C. lunata* (MC51)	0.2–0.3	55.56^e^	Moderate	0	0^a^	Avirulence	0.1–0.1	20.00^b^	Low
*C. lunata* (CA25)	0.2–0.4	55.56^e^	Moderate	0	0^a^	Avirulence	0.1–0.1	20.00^b^	Low
*C. lunata* (TB51)	0.1–0.2	33.33^c^	Low	0	0^a^	Avirulence	0.1–0.1	20.00^b^	Low
*C. gracilis* (CA22)	0.1–0.3	44.44^d^	Moderate	0	0^a^	Avirulence	0	0^a^	Avirulence
*C. gracilis* (CA64)	0.0–0.3	33.33^c^	Low	0	0^a^	Avirulence	0	0^a^	Avirulence
*S. bicolor* (MC14)	0.1–0.3	44.44^d^	Moderate	0	0^a^	Avirulence	0	0^a^	Avirulence
*S. bicolor* (TB21)	0.1–0.1	33.33^c^	Low	0	0^a^	Avirulence	0	0^a^	Avirulence
*S. bicolor* (TB43)	0.1–0.2	33.33^c^	Low	0	0^a^	Avirulence	0	0^a^	Avirulence
*A. verrucaria* (CA21)	0.1–0.1	33.33^c^	Low	0	0^a^	Avirulence	0.1–0.2	20.00^b^	Low
Control	0	0^a^	–	0	0^a^	–	0	0^a^	–

^a^Mean followed by the same letter are not significantly different (p < 0.05) according to Tukey’s test.

**Figure 5:**
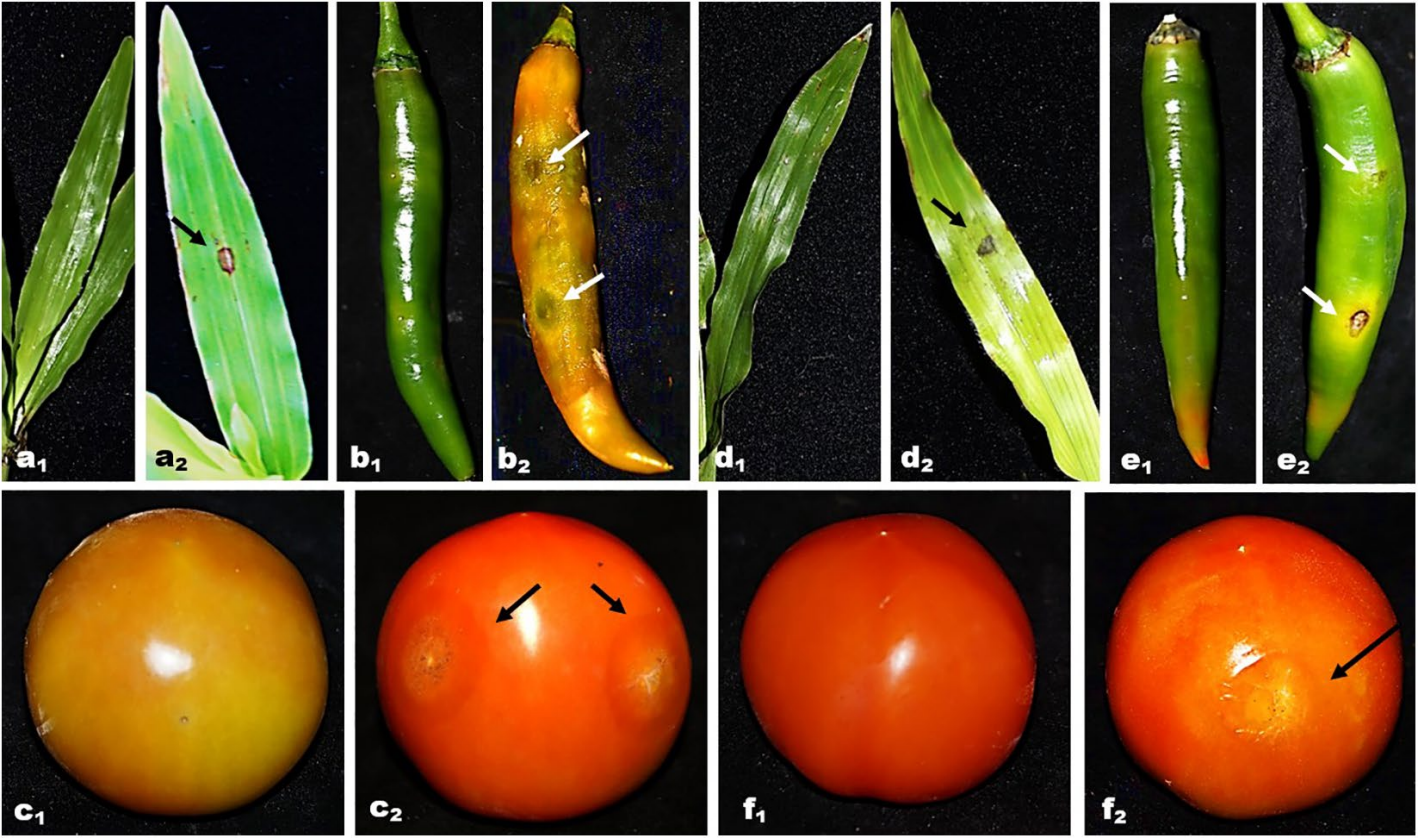
Pathogenicity test of several endophytic fungi on *A. compressus* leaves, chilli and tomato. Symptoms on wounded samples: (**a1**,**b1**,**c1**) Wounded control; (**a2**) Moderately severe rot lesion on wounded *A. compressus* leaves (*C. lunata* CA25); (**b2**) Highly severe rot lesion on chilli (*F. parceramosum* CA61); (**c2**) Moderately severe rot lesion on tomato (*C. endophyticum* ID23). Symptoms on unwounded samples: unwounded control (**d1**,**e1**,**f1**); (**d2**) Moderately severe rot lesion on *A. compressus* leaves (*C. lunata* MC51); (**e2**) Severe rot lesion on chilli (*F. parceramosum* CA52); (**f2**) Mildly severe rot lesion on tomato (*C. endophyticum* (ID45).

On wounded *A. compressus* leaves, a pinhead-sized lesion formed on the 5th day after inoculation, and a circular to irregular dark lesion (0.1–0.4 cm) was observed on the 7th day. The symptoms developed on the grass leaves were less severe than those on wounded chili and tomatoes (Table 1 and Fig. 5a2). The control *A. compressus* leaves, chili, and tomato fruits remained symptomless (Fig. 5a1,b1,c1). Pathogenicity tests on wounded chili and tomatoes indicated the ability of several fungal endophytes to infect both vegetable fruits. A rot lesion of 0.2–5.0 cm started to develop on the wounded site on the 4th day after inoculation (Fig. 5a2,b2,c2). Isolated fungal isolates from the infected tissues of *A. compressus* leaves, chili, and tomato showed the same morphological characteristics as the original isolates inoculated on the samples, confirming Koch’s postulates.

Among the fungal endophytes tested on the wounded *A. compressus* leaves, two isolates of *C. lunata* (MC51 and CA25) were the most virulent, with a DS of 55.56% (Table 1), followed by *C. gracilis* (CA22) with a DS of 44.44%. The other fungal endophytes were low-virulence isolates, with a DS of 33.33% (Table 1).

In wounded chili fruits, eight isolates of endophytic *F. parceramosum* showed moderate to very high virulence, with DS ranging from 46.67 to 100% (Table 1). Five isolates (CA61, CA52, ID22, MC35, and TB14) were categorized as highly virulent with a DS of 73.33–100% (Table 2). Endophytic *Colletotrichum* spp. tested on wounded chili showed a low-to-moderate degree of virulence. *Colletotrichum gigasporum* (MC31), *C. endophyticum* (ID45), and *C. siamense* (CA72) produced DS of 40–60%. A low degree of virulence was observed in *C. siamense* (ID31, MC52, and MC64), *C. gigasporum* (MC65), and *C. endophyticum* (ID23), with DS ranging from 20 to 26.67% (Table 1).

**Table 2:** Pathogenicity of endophytic fungi on unwounded *A. compressus* leaves, chilli and tomato on 7 day after inoculation.

Isolates	Grass leaves			Chilli			Tomato		
	Lesion length (cm)	Disease severity (%)	Degree of virulence	Lesion length (cm)	Disease severity (%)	Degree of virulence	Lesion length (cm)	Disease severity (%)	Degree of virulence
*C. endophyticum* (ID23)	0	0^a^	Avirulence	0	0^a^	Avirulence	0.0–1.5	20.00^d^	Low
*C. siamense* (ID31)	0	0^a^	Avirulence	0	0^a^	Avirulence	0.1–1.2	20.00^d^	Low
*C. siamense* (MC52)	0	0^a^	Avirulence	0	0^a^	Avirulence	0.0–0.5	6.67^b^	Low
*C. siamense* (CA72)	0	0^a^	Avirulence	0	0^a^	Avirulence	0.0–0.5	6.67^b^	Low
*C. endophyticum* (ID45)	0.0–0.1	22.22^b^	Low	0	0^a^	Avirulence	1.4–1.5	40.00^f^	Low
*F. parceramosum* (MC35)	0	0^a^	Avirulence	2.0–3.0	66.67^b^	High	0	0^a^	Avirulence
*F. parceramosum* (MC81)	0	0^a^	Avirulence	0	0^a^	Avirulence	0.6–1.0	33.33^e^	Low
*F. parceramosum* (CA52)	0	0^a^	Avirulence	2.5–3.0	73.33^c^	High	0	0^a^	Avirulence
*F. parceramosum* (CA61)	0.0–0.2	22.22^b^	Low	2.0–3.0	66.67^b^	High	0.0–0.1	6.67^b^	Low
*C. lunata* (ID34)	0.0–0.3	33.33^c^	Low	0	0^a^	Avirulence	0	0^a^	Avirulence
*C. lunata* (MC51)	0.1–0.3	44.44^d^	Moderate	0	0^a^	Avirulence	0	0^a^	Avirulence
*C. lunata* (CA25)	0.1–0.1	33.33^c^	Low	0	0^a^	Avirulence	0.0–0.1	13.33^c^	Low
Control	0	0^a^	–	0	0^a^	–	0	0^a^	–

^a^Mean followed by the same letter are not significantly different (p < 0.05) according to Tukey’s test.

The severity of the endophyte infection was lesser in wounded tomatoes than in wounded chili, with the endophytic fungi showing moderate to low degrees of virulence in the former. The highest DS of 60% was produced by *C. endophyticum* (ID23) (Fig. 5c2), followed by *C. siamense* (ID31) and *C. endophyticum* (ID45) (DS = 53.33%). Three isolates of *F. parceramosum* (MC81, CA52, and TB44) had a DS of 40%, whereas the other 15 isolates showed low virulence, with DS ranging from 20 to 33.33% (Table 2).

In the unwounded samples, 14 endophytic fungal isolates were non-pathogenic to *A. compressus* leaves, chili, and tomato fruits. In the infected samples, the rot lesions produced were generally similar to those on the wounded samples and started to appear on the 4th day after inoculation, becoming larger till the 7th day (2.0–3.0 cm). Control of unwounded samples are shown in Fig. 5d1,e1,f. Koch’s postulates were fulfilled as the same fungal isolates were reisolated from the inoculated sites.

On unwounded *A. compressus* leaves, five isolates produced rot lesions with a low-to-moderate degree of virulence. *Curvularia lunata* (MC51) had the highest DS (44.44%) and was categorized as a moderately virulent isolate (Table 2 and Fig. 5d2). Low virulence isolates included two isolates of *C. lunata* (ID34 and CA25) with a DS of 33.33%, and *F. parceramosum* (CA61) and *C. endophyticum* (ID45) with a DS of 22.22%.

Only three isolates of *F. parceramosum* (CA52, MC35, and CA61) were pathogenic to unwounded chili, producing rot lesions ranging from 2.0 to 3.0 cm. These isolates were categorized as highly virulent. *Fusarium parceramosum* (CA52) was the most virulent strain, with a DS of 73.33% (Table 2, Fig. 5e2). On unwounded tomatoes, eight isolates produced rot lesions ranging from 0.1 to 1.5 cm. *C. endophyticum* (ID45) (Fig. 5f2) had the highest DS (40%) with moderate virulence. Low virulence isolates with DS of 6.67–33.33% were *C. endophyticum* (ID23 and ID45), *F. parceramosum* (MC81), *C. siamense* (ID31, MC52, and CA72), *C. lunata* (CA25), and *F. parceramosum* (CA61) (Table 2). Generally, the symptoms produced in wounded samples were similar to those in unwounded samples. Koch’s postulates were fulfilled, as the same fungal isolates were re-isolated from the rot lesion.

## Discussion

The endophytic fungal isolates recovered from *A. compressus* leaves can be regarded as host generalists that are also present in other plant species. The isolates were identified as *F. parceramosum*, *C. siamense, C. gigasporum, C. endophyticum, C. lunata, S. bicolor, C. gracilis*, and *A. verrucaria* with potential pathogenicity. Therefore, endophytic fungi from *A. compressus* leaves may represent a group of latent plant pathogens.

The most common species recovered from *A. compressus* leaves were isolates of the *F. solani* species complex, phylogenetically identified as *F. parceramosum*. Endophytic *Fusarium* species are commonly isolated from Poaceae in the USA^22^. Endophytic *F. solani* has been isolated from various species of grasses in Malaysia^20,21^, Spain^23^, and Hungary^24^. These studies indicated that the *F. solani* species complex is part of the endophytic fungal assemblages in various species of Poaceae. Previously, *F. parceramosum* was known as the phylogenetic species FSSC18 reported as a rare human pathogen^25^. Later, *F. parceramosum* was recovered from plumbing systems^26^ and was recently associated with the cane blight of raspberry^27^.

A pathogenicity test of *F. parceramosum* showed a low degree of virulence on *A. compressus* leaves but a moderate to a high degree of virulence in tomato and chili, with severe rot symptoms. The findings indicated that endophytic *F. parceramosum* isolates from *A. compressus* were pathogenic to the grass host as well as other host plants, demonstrating that *A. compressus* harbors the plant pathogenic *F. parceramosum*. Species within the *F. solani* species complex have been recorded as pathogens on many plants and are associated with rot, wilt, canker, and dieback^28^.

Among the three endophytic *Colletotrichum* species, *C. siamense* and *C. endophyticum* were recovered from the grasses. *Colletotrichum siamense* has been isolated from dwarf Napier (*Pennisetum purpureum*) and lemon grass (*Cymbopogon citratus*)^29^. *Colletotrichum endophyticum* was first reported as an endophyte of dwarf Napier in northern Thailand^29^ and later reported in *Capsicum* fruit rot^30^. *Colletotrichum gigasporum* has not been reported in any grass species. Previously, the fungus was recovered from the healthy leaves of *Centella asiatica*, *Stylosanthes guianensis*, and *Coffea arabica*^31^.

In wounded samples, isolates of three endophytic *Colletotrichum* species tested were able to infect *A. compressus* leaves, chili, and tomato fruits with low to moderate virulence. Pathogenic *C. siamense* causes diseases in several plants, including leaf spot on macadamia^32^, black spot of strawberry^33^, fruit rot of chili^34^, and anthracnose on papaya^35^. *Colletotrichum gigasporum* has been identified as a causal pathogen of anthracnose in avocados and *Dalbergia odorifera*^36,37^. *Colletotrichum endophyticum* showed moderate virulence in chili and tomatoes but low virulence in *A. compressus*. The results suggested that chili and tomato might be the more preferred hosts for *C. endophyticum* than *A. compressus.* The findings of the present study are similar to those of de Silva et al.^38^, in which *Capsicum annuum* was the preferred host for *C. endophyticum* over *Pennisetum purpureum*.

*Curvularia lunata* is an endophyte and pathogen in various plants. Endophytic *C. lunata* was isolated from aromatic tall grass (*Cymbopogon caesius*) and barnyard grass weed (*Echinochloa glabrescens*)^39,40^ as well as from other plants, such as *Melia azedarach*^41^, *Phyllanthus amarus*^42^, and medical plants^43^.

Four isolates of endophytic *C. lunata* tested using the wounded method were only pathogenic to *A. compressus* leaves and tomato fruits. However, in unwounded samples, only two isolates were pathogenic to *A. compressus* leaves, and one isolate was pathogenic to tomato. In Poaceae, *C. lunata* was found to cause leaf spots on *A. compressus* and *Sorghum* *bicolor*^44,45^ as well as on corn^46–48^.

There is little information on *S. bicolor* and *C. gracilis* as endophytes and pathogens. Endophytic *S. bicolor* was the dominant species found in the sedge plant *Kobresia humilis*^49^, whereas endophytic *C. gracilis* was isolated from the roots of wild bananas^50^. *Albifimbria verrucaria* has been reported to be an endophyte in wild grapes^51^.

Endophytic *S. bicolor* and *C. gracilis* were pathogenic to *A. compressus* leaves with low to moderate virulence and were non-pathogenic to chili and tomato fruits. Most species of *Stagonospora* are associated with diseases in cereals^52^, and *S. bicolor* has been reported to cause leaf scorch in sugarcane^53,54^, which might explain why *S. bicolor* is not pathogenic to chili and tomato fruits. *C. gracilis* is an important pathogen in *Eucalyptus*^55^. *Albifimbria verrucaria* is pathogenic to *A. compressus* leaves and tomato fruit, with a low degree of virulence. *Albifimbria verrucaria* causes leaf spot in tomato^56^, soybean^57^, and spinach^58^.

Pathogenicity tests demonstrated that endophytic fungi from *A. compressus* can become pathogens and cause infection on wounded tissues. Wounds expedite the entry of isolates into the host, promoting infections. In the field, plants are exposed to environmental stress, herbivores, and insect feeding that cause wounding and pave the way for infection^59^.

Endophytic fungi can behave as latent and weak pathogens^60^. Potential pathogens of wheat and barley, including *Fusarium*, *Colletotrichum*, and *Stagonospora,* have been found in the perennial grass *Dactylis*^61–63^. Several endophytic fungi isolated from wild bananas are also latent pathogens^64^. In a study by Sakalidis et al.^65^, the endophytic *Lasiodiplodia theobromae* of baobab also behaved as a pathogen in its host plant. In the present study, all tested endophytic fungi were pathogenic on wounded leaves of *A. compressus*, suggesting that they have a latent ability to produce disease, as being opportunistic or facultative fungal endophytes. Some endophytic fungi can also infect chili and tomatoes. The result of the present study is in line with that of Kado^66^, who showed that pathogenic reactions may be observed when a latent pathogen was isolated from an apparently healthy host plant and introduced into a new host.

Fungal endophytes can transform into pathogens when the host encounters severe environmental stresses, such as extreme changes in moisture and temperature^15,66,67^. Under these conditions, imbalanced antagonism between the host plant and endophytes can result in diseases with visible symptoms^68^. Balance antagonism refers to balanced interactions between host defense mechanisms and fungal virulence, and when it is disturbed in favor of the fungus, the endophyte becomes pathogenic^15^. According to Saikkonen et al.^69^, endophytes can also become pathogens of other plants, depending on the balance between endophytism and pathogenicity of endophytes on various host plants.

The findings of the present study suggested that endophytic fungi from leaves of *A. compresses* may have pathogenic abilities, under stress conditions of the host plant, infecting not only the host plant but also other plants as well. These observations were based on an experiment with stressed organs of the plants. There is a possibility that endophytic fungi residing in *A. compressus* are facultative or opportunistic pathogens that act under certain conditions, especially when the host is under stress. Later due to increase in environmental stress, the endophyte may behave as pathogen. Moreover, the endophytic fungi recovered from *A. compressus* leaves in the present study have a wide host range which reflect their ability to infect other host plants particularly agricultural crops.

## Materials and methods

### Isolation of endophytic fungi

Healthy and symptomless *A. compressus* leaves were collected from three sites surrounding the main campus of Universiti Sains Malaysia (USM) in Penang, Malaysia. Twenty leaf samples were randomly collected and processed immediately after returning to the laboratory. Experimental research and field studies on the plants (either cultivated or wild), including the collection of the plant material are in compliance with relevant institutional, national, and international guidelines and legislation.

The leaves were surface sterilized with 70% alcohol for 1 min, followed by 5% sodium hypochlorite (NaOCl) for 1 min, and washed with sterile distilled water thrice for 1 min each. The leaves were blotted dry using sterile filter paper. Each sterilized leaf was cut into five segments and plated on potato dextrose agar (PDA; HiMedia Laboratory, Maharashtra, India). The sterilized leaves were incubated at room temperature (25 ± 1 °C) and observed daily for mycelial growth. To ensure the efficacy of the surface sterilization method, the leaf imprints^12^ and the last wash with the surface-sterilized solution were plated on PDA. The absence of fungal growth on these plates validated the effectiveness of the surface sterilization method, and the fungi obtained were proven to be endophytic.

The isolated endophytic fungi were sorted into genera based on their morphological characteristics and further identified using molecular markers, including transcribed spacer regions (ITS)^70^, glyceraldehyde-3-phosphate dehydrogenase (GAPDH)^71,72^, translation elongation factor 1-α (TEF-1α)^73,74^, β-tubulin (TUB)^75,76^, actin (ACT)^74^, and RNA polymerase II second largest subunit (RPB2)^77^ genes. The choice of marker depended on the fungal genera identified based on morphological characteristics.

### DNA extraction and PCR amplification

Genomic DNA of endophytic fungi was extracted using the Invisorb® Spin Plant Mini Kit (Stratec, Birkenfeld, Germany). Mycelia were harvested from potato dextrose broth and ground to a fine powder with liquid nitrogen using a sterile mortar and pestle. A total of 60 mg of fine mycelial powder was used for DNA extraction.

PCR reaction mixtures were prepared in a total volume of 50 µL containing 8 µL of 5X Green GoTaq^®^ Flexi Buffer, 8 µL of 25 mM MgCl_2_, 1 µL of 10 mM dNTP mix, 8 µL each of 5 µM RPB2 and 1 µM ACT and GAPDH as forward and reverse primers, respectively, deionized distilled water, 0.3 µL of 5 U/µL GoTaq^®^ DNA Polymerase (Promega, Madison, WI, USA), and 0.6 µL of DNA template.

Amplification was performed in a thermal cycler (Bio-Rad MyCycler PCR System version 1.065) with the following cycles: initial denaturation at 94 °C for 85 s, 35 cycles of denaturation at 95 °C for 35 s, annealing at 59.5 °C (RPB2), 58 °C (β-tubulin), and 61.5 °C (ACT and GAPDH) for 55 s, extension at 72 °C for 90 s, and a final extension at 72 °C for 10 min.

Agarose gel (1%) electrophoresis was used to detect PCR products in 1X Tris–borate-EDTA (TBE) buffer stained with FloroSafe DNA stain (Axil Scientific, Singapore). PCR products were sent to a service provider for DNA sequencing (NHK Bioscience Solutions, Malaysia).

### Molecular identification and phylogenetic analysis

After sequencing, a consensus sequence was formed by aligning the forward and reverse DNA sequences with ClustalW pairwise alignments using Molecular Evolution Genetic Analysis version 7 (MEGA7) software^78^. Consensus sequences were edited where necessary, and a BLAST search was performed against the GenBank database. For *Fusarium* isolates, a BLAST search was performed against the Fusarium-ID database.

Phylogenetic trees were constructed based on the combined sequences from multiple sequence alignments using MEGA7. A maximum likelihood (ML) tree was constructed with 1000 bootstraps replicates. We used a heuristic ML method, the nearest neighbor interchange (NNI), where the initial tree for ML is generated automatically. The best model for the ML tree was determined from a model search using five discrete gamma categories. The results showed that the Kimura 2 parameter model was the best.

### Pathogenicity test

Leaves of *A. compressus*, chili (*Capsicum annum*), and tomato (*Solanum lycopersicum*) were tested for pathogenicity. Healthy leaves of *A. compressus* were collected around the USM campus, while chilis and tomatoes were obtained from local supermarkets. A total of 26 representative isolates of endophytic fungi were selected for pathogenicity testing, consisting of eight isolates of *F. parceramosum* (ID22, 1D51, MC35, MC81, CA52, CA61, TB14, and TB44), five of *C. siamense* (ID31, MC52, MC64, and CA72), two of *C. gigasporum* (MC31 and MC65), two of *C. endophyticum* (ID23 and ID45), four of *C. lunata* (1D34, MC52, CA25, and TB51), three of *S. bicolor* (MC14, TB21, and TB43), two of *C. gracilis* (CA22 and CA64), and one of *A. verrucaria* (CA21).

The detached leaf method was applied in the pathogenicity test^79^ using mycelial plugs on two groups: wounded and unwounded leaves. The leaves of *A. compressus* and tomato were surface-sterilized using 70% ethanol, soaked in 70% ethanol for 3 min, and rinsed with sterile distilled water three times. The samples were then air-dried under laminar flow. Samples were wounded by pricking using a sterile scalpel. Mycelial plugs (5 mm) were cut using a cork borer at the edges of actively sporulated colonies and inoculated on the surfaces of wounded and unwounded samples. Mycelial plugs inoculated on the surface of the sample were covered with moistened cotton wool and cellophane tape.

The inoculated samples were incubated in a clear rounded container (24 cm^2^) at room temperature (25 ± 1 °C) for 7 days. Each experiment was performed in triplicate and repeated twice. Disease development was observed every day, and lesion size was measured on the 7th day.

Tissues from the infected samples were isolated on PDA plates and morphologically identified. Koch’s postulates were fulfilled if the fungal isolates from infected samples were morphologically similar to the original inoculated isolates.

### Disease assessment

Disease development was observed daily, and lesion size was measured on day 7 after inoculation. The severity of the rot lesion formed on infected samples was estimated based on the disease scale by Chavan and Tawade^80^ with some modifications. The disease severity scales used to assess the infection on *A. compressus* leaves were as follows: 0 (no symptoms, rot lesion = 0 cm); 1 (slightly severe, rot lesion = 0.1–0.2 cm); 2 (moderately severe, rot lesion = 0.3–0.4 cm); and 3 (highly severe, rot lesion =  > 0.5 cm). For disease assessment of infection in chili and tomato, the disease scales applied were as follows: 0 (no obvious symptom, rot lesion = 0 cm); 1 (slightly severe, rot lesion = 0.1–0.9 cm); 2 (mildly severe, rot lesion = 1.0–1.9 cm); 3 (moderately severe, rot lesion = 2.0–2.9 cm); 4 (severe, rot lesion = 3.0–3.9 cm), and 5 (highly severe =  > 4 cm).

To determine the virulence level, the percentage of disease severity (DS) was calculated according to the formula by Cooke^81^. Analysis of variance (ANOVA) with Tukey’s test (p < 0.05) was used to analyze the data using SPSS statistical software version 26, Armonk, NY: IBM Corp.

The disease severity (DS) calculated as DS = [Σ (a × b)/NZ] × 100%, where Σ (a × b) = the sum of the infected leaves and fruits and their corresponding score scale, N is the total number of sampled leaves and fruits, and Z is the highest value on the disease scale. Based on the DS percentage, the degree of virulence was determined according to Charoenporn et al.^82^ with some modifications: avirulence (DS = 0.00%), low virulence (DS ≤ 35.00%), moderate virulence (DS > 36.00–60.00%), high virulence (DS > 61.00–80.00%), and very high virulence (DS > 80%).

## Supplementary Information

Supplementary Information.

## Data Availability

The datasets used and/or analysed during the current study available from the corresponding author on reasonable request. The sequences analysed during this study are available in the GenBank: https://www.ncbi.nlm.nih.gov/genbank/ (the accession numbers are indicated in Table 1).
