# Supplementary material for: Molecular identification of endophytic fungi in lawn grass (Axonopus compressus) and their pathogenic ability
Source: Sci Rep. 2023 Mar 14;13:4239. doi: 10.1038/s41598-023-31291-7 (PMC10015033; doi:10.1038/s41598-023-31291-7)
Supplement: Supplementary file 1 — Supplementary Information. [file 41598_2023_31291_MOESM1_ESM.pdf]

Supplementary Table 1. Molecular identification of endophytic fungi isolated from leaves of *A. compressus* and GenBank accession number

Isolates	Markers and GenBank Accession Number						
	TEF-1α	ITS	β-tubulin	GAPDH	ACT	RPB2	Similarity (%)
Fusarium solani species complex (*Fusarium parceramosum*)							
CA32	ON244471	-	ON244473	-	-	ON244487	99-100%
CA43	ON244472	-	ON244474	-	-	ON244488	99-100%
CA52	ON244470	-	ON244475	-	-	ON244489	99-100%
CA61	ON244469	-	ON244476	-	-	ON244490	99-100%
ID22	ON244459	-	ON244477	-	-	ON244491	99-100%
ID43	ON244460	-	ON244478	-	-	ON244492	99-100%
ID51	ON244461	-	ON244479	-	-	ON244493	99-100%
MC35	ON244462	-	ON244480	-	-	ON244494	99-100%
MC74	ON244463	-	ON244481	-	-	ON244495	99-100%
MC75	ON244464	-	ON244482	-	-	ON244496	99-100%
MC81	ON244465	-	ON244483	-	-	ON244497	99-100%
MC84	ON244466	-	ON244484	-	-	ON244498	99-100%
TB14	ON244467	-	ON244486	-	-	ON244499	99-100%
TB44	ON244468	-	ON244485	-	-	ON244500	99-100%
Colletotrichum siamense							
ID21	-	ON194053	ON755279	ON755291	ON755303	-	99-100%
ID31	-	ON194055	ON755280	ON755292	ON755304	-	99-100%
MC52	-	ON194056	ON755281	ON755294	ON755306	-	99-100%
MC61	-	ON194057	ON755282	ON755293	ON755305	-	99-100%
MC64	-	ON194058	ON755283	ON755297	ON755310	-	99-100%
MC72	-	ON194059	ON755284	ON755295	ON755308	-	99-100%
MC82	-	ON194060	ON755285	ON755296	ON755309	-	99-100%
CA72	-	ON194061	ON755286	ON755298	ON755307	-	99-100%
Colletotrichum gigasporum							
MC31	-	ON194063	ON755289	ON755301	ON755311	-	99-100%
MC65	-	ON194064	ON755290	ON755302	ON755312	-	99-100%

Colletotrichum endophyticum							
ID23	-	ON194054	ON755287	ON755299	ON755313	-	99-100%
ID45	-	ON194071	ON755288	ON755300	ON755314	-	99-100%
Curvularia lunata							
CA25	-	ON194149	-	OP312085	-	-	99-100%
CA51	-	ON194150	-	OP312086	-	-	99-100%
ID13	-	ON194151	-	OP312087	-	-	99-100%
ID34	-	ON194152	-	OP312088	-	-	99-100%
ID41	-	ON194153	-	OP312089	-	-	99-100%
MC51	-	ON194154	-	OP312090	-	-	99-100%
TB51	-	ON194155	-	OP312091	-	-	99-100%
Stagonospora bicolor							
MC14	-	ON194254	-	-	-	-	99-100%
TB21	-	ON194255	-	-	-	-	99-100%
TB24	-	ON194256	-	-	-	-	99-100%
TB32	-	ON194257	-	-	-	-	99-100%
TB43	-	ON194258	-	-	-	-	99-100%
Calonectria gracilis							
CA22	-	ON197176	-	-	-	-	99%
CA62	-	ON197177	-	-	-	-	99%
CA64	-	ON197178	-	-	-	-	99%
Albifimbria verrucaria							
CA21	-	ON197179	-	-	-	-	98%

Suppplementary Table 2: BLAST search results of endophytic *F. solani* isolates recovered from leaves of *A. compressus*

	**Markers (% similarity)**			
**Isolate**	**TEF-1α GenBank**	**TEF-1α Fusarium-ID**	**RPB2 Genbank**	**RPB2 Fusarium-ID**
CA32	1) *F. solani* FRC S1933L (99.84%)<br>2) *F. solani* ICMP:2093 (98.91)	1) *F. liriodendri* (97.40%)<br>2) *F. parceramosum* (97.05%)	1) *F. parceramosum* (99.62%)<br>2) *F. liriodendri* (99.62%)	1) *F. liriodendri* (99.67%)<br>2) *F. perseae* (97.41%)
CA43	1) *F. solani* FRC S1933L (99.84%)<br>2) *F. solani* ICMP:2093 (98.91)	1) *F. liriodendri* (97.40%)<br>2) *F. parceramosum* (97.05%)	1) *F. parceramosum* (99.62%)<br>2) *F. liriodendri* (99.62%)	1) *F. liriodendri* (99.67%)<br>2) *F. perseae* (97.41%)
CA52	1) *F. solani* FRC S1933L (99.84%)<br>2) *F. solani* ICMP:2093 (98.91)	1) *F. liriodendri* (97.40%)<br>2) *F. parceramosum* (97.05%)	1) *F. parceramosum* (99.62%)<br>2) *F. liriodendri* (99.62%)	1) *F. liriodendri* (99.67%)<br>2) *F. perseae* (97.41%)
CA61	1) *F. solani* FRC S1933L (99.84%)<br>2) *F. solani* ICMP:2093 (98.91)	1) *F. liriodendri* (97.40%)<br>2) *F. parceramosum* (97.05%)	1) *F. parceramosum* (99.62%)<br>2) *F. liriodendri* (99.62%)	1) *F. liriodendri* (99.67%)<br>2) *F. perseae* (97.41%)
ID22	1) *F. solani* FRC S1933L (99.84%)<br>2) *F. solani* ICMP:2093 (98.91)	1) *F. liriodendri* (97.40%)<br>2) *F. parceramosum* (97.05%)	1) *F. parceramosum* (99.62%)<br>2) *F. liriodendri* (99.62%)	1) *F. liriodendri* (99.67%)<br>2) *F. perseae* (97.41%)

ID43	1) *F. solani* FRC S1933L (99.84%)<br>2) *F. solani* ICMP:2093 (98.91)	1) *F. liriodendri* (97.40%)<br>2) *F. parceramosum* (97.05%)	1) *F. parceramosum* (99.62%)<br>2) *F. liriodendri* (99.62%)	1) *F. liriodendri* (99.67%)<br>2) *F. perseae* (97.41%)
ID51	1) *F. solani* FRC S1933L (99.84%)<br>2) *F. solani* ICMP:2093 (98.91)	1) *F. liriodendri* (97.40%)<br>2) *F. parceramosum* (97.05%)	1) *F. parceramosum* (99.62%)<br>2) *F. liriodendri* (99.62%)	1) *F. liriodendri* (99.67%)<br>2) *F. perseae* (97.41%)
MC35	1) *F. solani* FRC S1933L (99.84%)<br>2) *F. solani* ICMP:2093 (98.91%)	1) *F. liriodendri* (97.40%)<br>2) *F. parceramosum* (97.05%)	1) *F. parceramosum* (99.62%)<br>2) *F. liriodendri* (99.62%)	1) *F. liriodendri* (99.67%)<br>2) *F. perseae* (97.41%)
MC74	1) *F. solani* FRC S1933L (99.84%)<br>2) *F. solani* ICMP:2093 (98.91)	1) *F. liriodendri* (97.40%)<br>2) *F. parceramosum* (97.05%)	1) *F. parceramosum* (99.62%)<br>2) *F. liriodendri* (99.62%)	1) *F. liriodendri* (99.67%)<br>2) *F. perseae* (97.41%)
MC75	1) *F. solani* FRC S1933L (99.84%)<br>2) *F. solani* ICMP:2093 (98.91)	1) *F. liriodendri* (97.40%)<br>2) *F. parceramosum* (97.05%)	1) *F. parceramosum* (99.62%)<br>2) *F. liriodendri* (99.62%)	1) *F. liriodendri* (99.67%)<br>2) *F. perseae* (97.41%)
MC81	1) *F. solani* FRC S1933L (99.84%)<br>2) *F. solani* ICMP:2093 (98.91)	1) *F. liriodendri* (97.40%)<br>2) *F. parceramosum* (97.05%)	1) *F. parceramosum* (99.62%)<br>2) *F. liriodendri* (99.62%)	1) *F. liriodendri* (99.67%)<br>2) *F. perseae* (97.41%)
MC84	1) *F. solani* FRC S1933L (99.84%)	1) *F. liriodendri* (97.40%)	1) *F. parceramosum* (99.62%)	1) *F. liriodendri* (99.67%)

	2) *F. solani* ICMP:2093 (98.91)	2) *F. parceramosum* (97.05%)	2) *F. liriodendri* (99.62%)	2) *F. perseae* (97.41%)
TB14	1) *F. solani* FRC S1933L (99.84%)<br>2) *F. solani* ICMP:2093 (98.91)	1) *F. liriodendri* (97.40%)<br>2) *F. parceramosum* (97.05%)	1) *F. parceramosum* (99.62%)<br>2) *F. liriodendri* (99.62%)	1) *F. liriodendri* (99.67%)<br>2) *F. perseae* (97.41%)
TB44	1) *F. solani* FRC S1933L (99.84%)<br>2) *F. solani* ICMP:2093 (98.91)	1) *F. liriodendri* (97.40%)<br>2) *F. parceramosum* (97.05%)	1) *F. parceramosum* (99.62%)<br>2) *F. liriodendri* (99.62%)	1) *F. liriodendri* (99.67%)<br>2) *F. perseae* (97.41%)

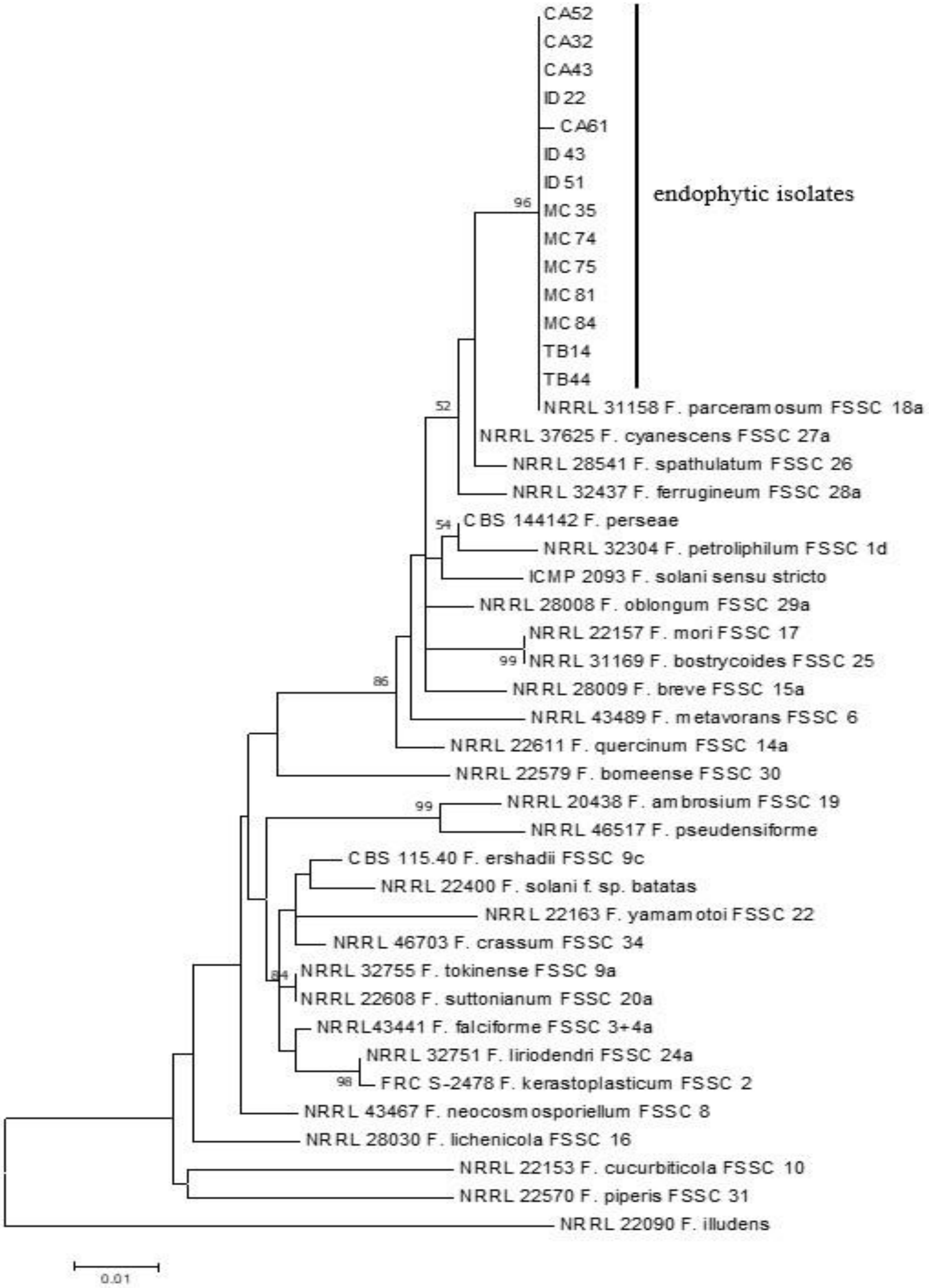

Supp. Figure 1: Maximum-likelihood tree inferred from TEF-1α sequences of endophytic *F. solani* species complex isolates isolated from *A. compressus* leaves.
